# The EpiFusion Analysis Framework for joint phylodynamic and epidemiological analysis of outbreak characteristics

**DOI:** 10.12688/f1000research.162719.1

**Published:** 2025-03-28

**Authors:** Ciara Judge, Oliver Brady, Sarah Hill

**Affiliations:** 1 Department of Infectious Disease Epidemiology and Dynamics, London School of Hygiene and Tropical Medicine Faculty of Epidemiology and Population Health, London, England, UK; 2 Centre for Mathematical Modelling of Infectious Diseases, London School of Hygiene and Tropical Medicine, London, UK; 3 Department of Pathobiology and Population Sciences, The Royal Veterinary College, Hatfield, England, UK

**Keywords:** research software, phylodynamics, epidemiology, joint inference, effective reproduction number, infectious disease, modelling, R, java, particle filtering

## Abstract

The fields of epidemiology and viral phylodynamics share the ultimate goal of disease control, but concepts, methodologies and data employed by each differ in ways that confer complementary strengths and different areas of weakness. We recently introduced EpiFusion, a model for joint inference of outbreak characteristics using phylogenetic and case incidence data via particle filtering and demonstrated its usage to infer the effective reproduction number of simulated and real outbreaks. Here we provide a series of vignettes demonstrating data analysis using the EpiFusion Analysis Framework, consisting of the R package EpiFusionUtilities and the Java program in which the model is implemented, including an example using a new feature incorporated since EpiFusion’s last description: the option to provide a phylogenetic tree posterior as the phylogenetic data input to the program. By outlining these examples, we aim to improve the usability of our model, and promote workflow reproducibility and open research.

## Introduction

Implementing mathematical models of infectious disease outbreak characteristics using computational tools is an important aspect of public health research. ^1, 2^ Tools are often distributed as packages or libraries in popular programming languages such as R, Python or Julia, or as standalone executable software. ^3– 6^ Distributing and documenting workflows is important for the purposes of reproducible research, and to enable appropriate implementation of epidemiological models. ^7– 9^ Previously we outlined EpiFusion, a novel method for modelling infectious disease outbreak characteristics conditioned on case incidence and phylogenetic trees using particle filtering, and validated its usage to infer infection trajectories and the effective reproduction number $R_{t}.$ ^10^ Here, we present the EpiFusion Analysis Framework, consisting of this EpiFusion model implemented as a Java command line tool, and the EpiFusionUtilities R package for data and output processing.

EpiFusion consists of a ‘single process model, dual observation model’ particle filtering structure, where particles simulate outbreak trajectories and characteristics through time (process model) and are evaluated against phylodynamic and epidemiological data at resampling steps (observation models). EpiFusion uses case incidence and phylogenetic tree(s) as its data input but can also be run with either data type alone. The force of infection over time β is fit within the particle filter, and the recovery rate, case sampling rate, and genomic sequence sampling rate ( γ , ϕ and ψ respectively) are fit via Markov Chain Monte Carlo (MCMC). Further information including model theory and validation are provided in Ref. 10. The EpiFusion model is packaged as a Java ^11^ command line tool and takes eXtensible Markup Language (XML) files ^12^ as input.

In this article we provide instruction on implementing the recommended workflow (‘The EpiFusion Analysis Framework’) using an EpiFusion model. This includes data pre-processing, parameterisation, and eventual output parsing from within an R session, using the R package EpiFusionUtilities ( https://github.com/ciarajudge/EpiFusionUtilities). We also demonstrate start-to-finish use cases for complete analysis of two outbreak datasets using EpiFusion and EpiFusionUtilities.

## Methods

### Operation

EpiFusion is implemented as an open-source Java software (version 8 or later) and can be used as a command line tool or from within the EpiFusionUtilities R package ( https://ciarajudge.github.io/EpiFusionUtilities/). The latest stable version of the program is available for download under Releases on the project Github repository ( https://github.com/ciarajudge/EpiFusion/releases). The source code for the latest development version is also available at this repository for users who wish to clone the repository and compile the program from source. EpiFusion can be called using its full file path, or from any working directory on your system by creating a symbolic link *(Appendix 1).*

EpiFusionUtilities is implemented as an open source R ^13^ (version 3.5.0 or later) package and is available to install from Github using the R package devtools ^14^:

*# install from Github*
devtools**::install_github**("https://github.com/ciarajudge/EpiFusionUtilities")



The key steps of the EpiFusion Framework workflow are outlined below in brief in the ‘Implementation’ section.

### Implementation

**Figure 1.**
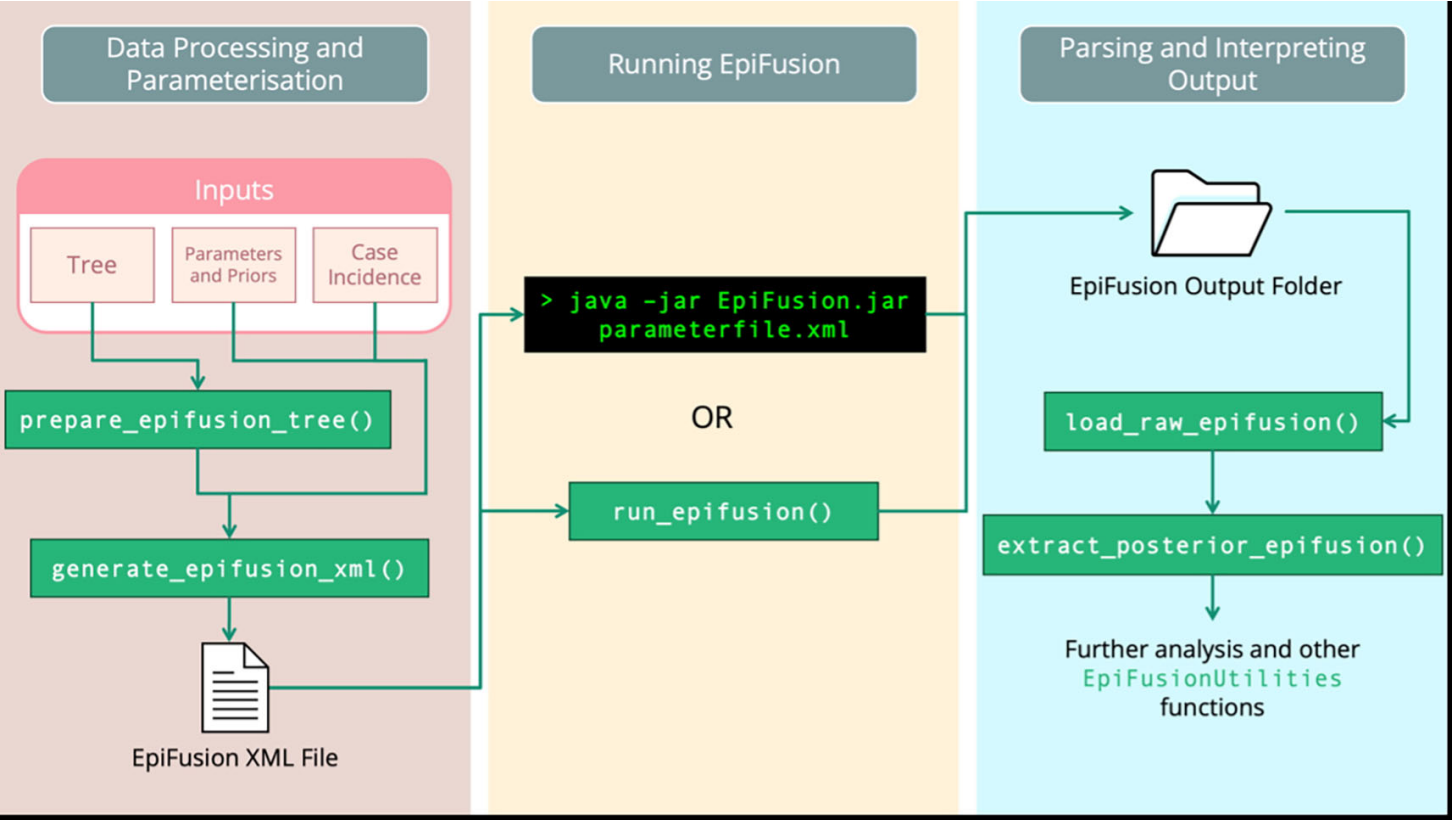
Recommended EpiFusion Framework workflow, using EpiFusionUtilities functions (green) to prepare data and parse results.

The EpiFusion Analysis Framework consists of three main steps: (i) Data Processing and Parameterisation (ii) Running EpiFusion and (iii) Parsing and Interpreting the output ( Figure 1). All three steps can be carried out using EpiFusionUtilities, but it is also possible to manually assemble an XML input file and run EpiFusion from the command line using an executable jar file.

**Data processing and parameterisation**

*EpiFusion XML*

EpiFusion uses input files written in eXtensible Markup Language (XML) to provide all data and parameterisation to the program. These files contain Loggers, Data, Analysis, Model, Parameters and Priors sections where various aspects of the model and analysis may be specified ( Table 1). A full breakdown of the options available within each section is included in the Supplementary Information *(Appendix 2).*

**Table 1.** Main sections of EpiFusion XML parameter file structure.

XML section	Description
*Loggers*	Provides detail on the program output, including specifying the file path of the output folder that should be created, and the frequency at which the program both logs the state of the MCMC to the output files and prints to the console.
*Data*	Provides the case incidence data and/or a phylogenetic tree or trees. These can be supplied either directly within the XML document or by providing full file paths to files containing the data)
*Analysis*	Parameterises the method for fitting the infectivity parameter beta - this is the daily probability per infectious individual of infecting another individual.
*Model*	Allows further customisation of the EpiFusion model structure. Currently, this only includes specification of the epidemiological observation model.
*Parameters*	Specifies many assorted parameters for the model, for example number of MCMC steps per chain, number of MCMC chains, and number of particles in the particle filter.
*Priors*	Prior distribution specification for parameters to be fit via particle MCMC. A range of distribution options are available, including: Normal, Truncated Normal, Poisson, Uniform, Uniform Discrete, Beta and Fixed (if a parameter should be fixed to a specific value and not inferred via MCMC).

*Assembling parameter XML files*

EpiFusion XML files may either be populated manually using templates available at the EpiFusion Github repository under examples, or created using a number of functions in the EpiFusionUtilities package.

The first step of processing case incidence or tree data for EpiFusion input is to select an ‘index date’. This date should equate approximately to the ‘date of origin’ of the outbreak under consideration, i.e. the suspected date of the first infection/importation to the geographical or demographic system under study (day ‘0’). The index date should fall before any birth events (internal nodes / branching events) in the tree or observed epidemiological cases. This date is provided to the processing functions to enable the case and incidence data to be rooted in numerical time units. All trajectory samples will assume that the outbreak originated with one individual becoming infected on the index date. All times in the EpiFusion input and output will be in relation to this date and measured in days.

index_date <- **as.Date**("2024-01-01")



If there is uncertainty in the date of origin of the outbreak we recommend setting the index date to earlier than the estimated date. The resulting trajectories will likely demonstrate high uncertainty during the earliest days of the outbreak, until the point of the time series at which some data becomes available.

To prepare a tree or posterior set of trees for EpiFusion, pass an S3 phylo or multiPhylo object in R to the prepare_epifusion_tree () function (we recommend any standard phylogenetics R package to manipulate these objects ^15, 16^). This function processes a phylogenetic tree (or trees) and writes to a file, which you can specify in the arguments of the function (the default is ‘./processedtree.tree’). It is also necessary to pass the date of the last sample in the tree(s). This function adds node and leaf labels to the tree string that correspond to their time in days after the index date.

**prepare_epifusion_tree**(tree,
                       index_date,
                       last_sequence_date,
                       "Data/Processed/processed_fixed_tree.tree")



To generate an EpiFusion XML file from within an R session, the generate_epifusion_XML () function may be used. This function populates a template XML file (included with the EpiFusionUtilities package) with the phylogenetic and/or case incidence data, and has default settings for parameters and priors which can also be changed by providing new values in the arguments of the function. For example below, we pass a case incidence data frame (consisting of two columns: a Date column named Date, with a numeric column Cases consisting of the number of cases reported on the corresponding date) and tree to the function. We also specify that we will sample from the MCMC chain every 100 steps, set our output folder path to output_files, and adjust the number of particles in the particle filter to 300. This creates a file in our working directory, epifusion_input.xml, which is ready to pass to EpiFusion.

logger_information <- **list**(fileBase = "output_files", logEvery = 100)
parameters_to_adjust <- **list**(numParticles = 300)

**generate_epifusion_XML**(tree = "Data/Processed/processed_fixed_tree.tree",
                       case_incidence = case_incidence,
                       index_date = index_date,
                       loggers = logger_information,
                       parameters = parameters_to_adjust,
                       xml_filepath = "epifusion_input.xml")



We include guidance on parameterisation and setting reasonable parameters and priors in the Supplementary Information alongside our more detailed description of EpiFusion XML *(Appendix 2).*

**Running EpiFusion**

EpiFusion can be run directly from the command line by calling an executable Java Archive (JAR) file using the following syntax. Here EpiFusion.jar is the file path to the executable file (i.e. in this example, the file is present in the working directory) and epifusion_input.xml is the file path to the parameter XML file (also present in the working directory for this example):

java -jar EpiFusion.jar epifusion_input.xml



Alternatively, it is possible to run EpiFusion from inside an R session with the EpiFusionUtilities function run_epifusion(). An installation of Java is still required.

**run_epifusion**("epifusion_input.xml")



**Interpreting output**

EpiFusion creates a directory within the working directory that corresponds to the file path of the fileBase parameter in your EpiFusion xml file. For each MCMC chain, EpiFusion will create the following output files:

• **betas**: .csv file where each row is a daily trajectory of infectivity β sampled from the MCMC
• **trajectories:** .csv file where each row is a daily trajectory sampled from the MCMC of the number of individuals infected over time
• **params:** .txt file where each column is an MCMC parameter, and each row is an MCMC sample
• **likelihoods:** .txt file of the posterior likelihoods calculated at each MCMC step
• **acceptance:** .txt file where each line logs the acceptance rate of steps between MCMC samples
• **completed:** .txt file where each line logs if the particle filter step was completed or quit due to particle depletion
• **cuminfections:** .txt file where each row is a trajectory of cumulative infections per day sampled from the MCMC
• **positivetests (only for combined or epi-only analyses):** .csv file where each row is simulated case incidence by the model which was compared to the observed case incidence. This is provided at the same resolution as the observed case incidence, i.e. there will be one column per case incidence data point.

EpiFusion will also save a copy of the parameter file used to the output folder, to record which parameters were used, and a file called ‘timings.txt’ with the runtime in nanoseconds.

It is possible to process this raw output manually, but EpiFusionUtilities provides a number of functions to do this from within R. The following functions load the raw output into an R object, plot the likelihood trace for each MCMC chain to enable inspection to decide what proportion of samples from each chain to discard as burn-in, and finally extract the posterior samples from each chain with a given proportion discarded and combine them into a single posterior while assessing convergence. To select an appropriate burn-in proportion, we recommend inspecting the likelihood and parameter trace plots to assess the point at which samples along the x-axis form a stationary distribution randomly sampled from the same region of the y-axis. ^17^ It is also possible to test multiple extractions extract_posterior_epifusion() with different burn-in proportions, and inspect the gelman-rubin statistics of the parameters, which indicate convergence if below 1.015. ^18^

raw_output <- **load_raw_epifusion**("output_files/")
**plot_likelihood_trace**(raw_output)
full_posterior <- **extract_posterior_epifusion**(raw_output, 0.1)



Below we demonstrate the implementation of this workflow using the EpiFusion executable and EpiFusionUtilities to analyse data from a small simulated outbreak.

## Use cases

### Description of the simulated datasets

**Figure 2.**
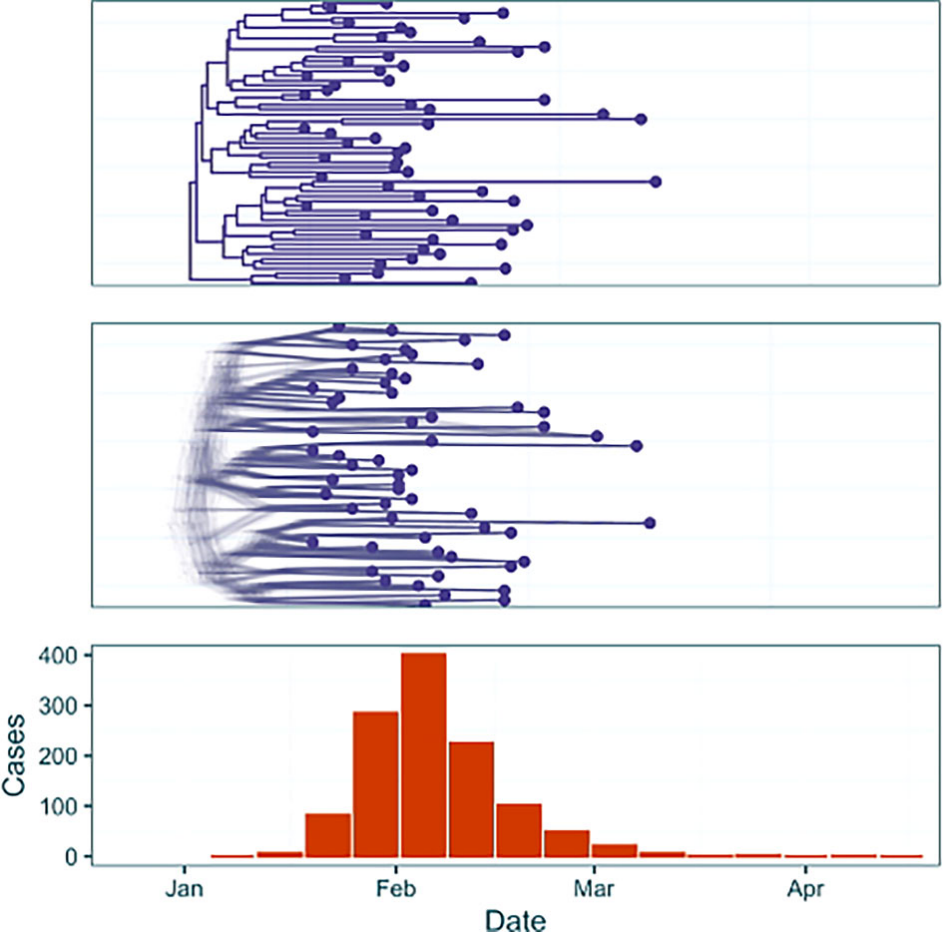
Data from the baseline outbreak simulated using ReMaster and BEAST 2.7.3. The dataset contains both a summary phylogenetic tree (top) and tree posterior samples (middle), and weekly case incidence (orange).

**Figure 3.**
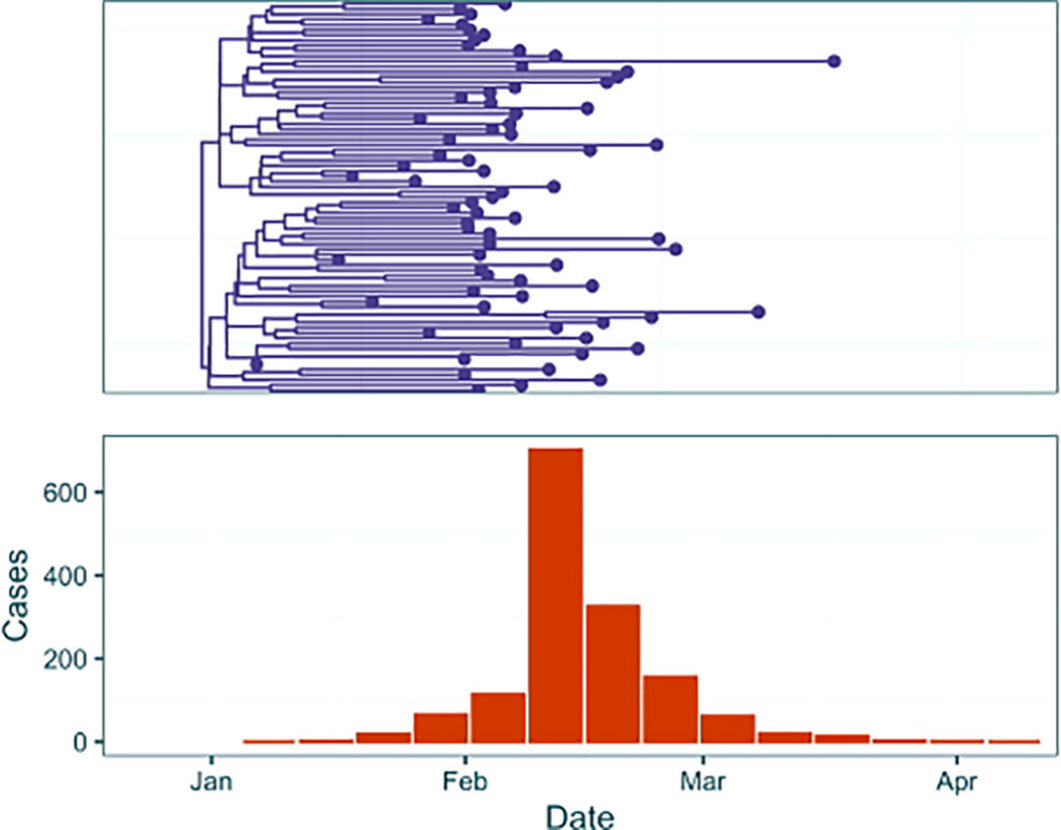
Data from outbreak with a step-change increase in sampling simulated using ReMaster.

We demonstrate three analyses on two simulated outbreak datasets ( Figures 2, 3). First we address a simple outbreak that lasts approximately three months with constant sampling effort that captures a single epidemic peak (‘baseline’). In the subsection ‘Full Framework Workflow’ we will model this outbreak using case incidence data in conjunction with a fixed time-scaled phylogenetic tree. Next, in ‘Phylogenetic Uncertainty’ we will model the same outbreak whilst examining the effect of phylogenetic uncertainty by using a tree posterior generated using a BEAST analysis of genomic samples simulated from the outbreak. Finally, in ‘Introducing Rate Changes’, we examine an outbreak with similar transmission dynamics but where initial minimal sampling of cases and sequences is followed by a sharp increase in the sampling rate on February 5 ^th^ 2024, and demonstrate how to parameterise this in an EpiFusion model. This example attempts to capture the challenges that often accompany modelling real-world outbreaks, where circumstances may evolve as an outbreak progresses (e.g. changes in case definitions affecting sampling rates, or upscaling of PCR diagnostics in response to an emerging infectious disease).

To generate the data, outbreak trajectories, and resulting weekly case incidence and a transmission tree of cases were simulated using ReMaster. ^19^ To give a simulated phylogenetic tree of ‘sequenced samples’ from the outbreak the transmission trees were downsampled, as typically only a small proportion of cases are sequenced in even heavily sampled outbreak settings. ^20, 21^ For the baseline outbreak genomic sequences were simulated in R from this phylogenetic tree using the function simSeq() from the R package phangorn. ^16^ These sequences were used to generate a tree posterior using BEAST 2.7.3 ^22^ with a Birth Death Skyline model, ^23^ under a strict clock and JC69 substitution model.

The date of origin of each outbreak was arbitrarily chosen as January 1st 2024 and the case data and tree leaves were labelled accordingly. The final resulting data inputs for analysis in EpiFusion consisted of a file with a fixed time-scaled phylogenetic tree, a tree posterior file generated from sequences simulated from the outbreak, and a csv file with dated counts of weekly incidence. These raw data files are provided alongside the code below in the article repository ( https://github.com/ciarajudge/EpiFusion_Vignettes). The data is also provided directly as part of the EpiFusionUtilities package, and can be loaded directly into R using the functions baseline_dataset and sampling_dataset.

### Full framework workflow

In this example, we will use the baseline dataset to show a full workflow using the EpiFusion Framework: (i) data preparation (ii) prior and parameter specification (iii) running EpiFusion (iv) parsing and plotting output.

**Data preparation**

First we load and inspect the data for this example using the EpiFusionUtilities function baseline_dataset(). This function loads a data frame with weekly case incidence (formatted with two columns, Cases and Date, where Cases = the number of epidemiological cases reported on the corresponding Date), a time-scaled phylogenetic tree, and samples from a tree posterior (with 50% burn-in removed) from a BEAST analysis which we will use in a later section (Phylogenetic Uncertainty).

**baseline_dataset**()

**print**(baseline_caseincidence[1**:**5,])
##   Cases       Date
## 1     0 2024-01-08
## 2     6 2024-01-15
## 3    83 2024-01-22
## 4   285 2024-01-29
## 5   401 2024-02-05
**print**(baseline_tree)
##
## Phylogenetic tree with 59 tips and 58 internal nodes.
##
## Tip labels:
## sequence1|2024-01-25, sequence2|2024-01-30, sequence3|2024-02-12, sequence4|2024-01-30, sequence5|2024-02-04, sequence6|2024-02-17, …
## Node labels:
## node_1, node_4, node_14, node_97, node_106, node_158, …
##
## Rooted; includes branch lengths.
**print**(baseline_treeposterior)
## 200 phylogenetic trees



Next we set two date objects: the ‘index date’, or the earliest date from which we will model the outbreak origin date, and the date of sampling of the last observed sequence from the dataset. Whilst for this example we know (through the simulation process) that the outbreak origin was the 1st of January 2024, it is good practice to set the index date to some time before the date that we suspect the outbreak began in the location represented by our case and phylogenetic data, to ensure the outbreak dynamics of are fully captured.

index_date <- **as.Date**("2023-12-26")
last_sequence <- **as.Date**("2024-03-10")



To prepare the tree objects for EpiFusion we can use the prepare_epifusion_tree function from EpiFusionUtilities. This function processes the tree(s) for input to EpiFusion and writes them to the provided file path. In the case where a single summary tree is provided to this function it also returns the processed tree as an R phylo object, which here we reassign to the variable fixed_tree.

fixed_tree <- **prepare_epifusion_tree**(baseline_tree, index_date, last_sequence, "Data/Processed/baseline_fixed_tree.tree")



**Definition of parameters**

We will create an EpiFusion XML file using the generate_epifusion_xml function from EpiFusionUtilities. This function populates the below XML template with our data and creates a new file. It is often necessary to adjust some other parameters from their default values in this template. This can be achieved by providing additional arguments to the generate_epifusion_xml function, which we demonstrate below.

<?xml version="1.0" encoding="UTF-8"?>
<EpiFusionInputs>
  <loggers>
    <fileBase>FILESTEM</fileBase>
    <logEvery>10</logEvery>
  </loggers>
  <data>
    <incidence>
      <incidenceVals>INCIDENCE</incidenceVals>
      <incidenceTimes type="exact">INCIDENCETIMES</incidenceTimes>
   </incidence>
   <tree>
     <treePosterior></treePosterior>
   </tree>
   <epicontrib>0.5</epicontrib>
   <changetimes>0</changetimes>
  </data>
  <analysis>
    <type>looseformbeta</type>
    <startTime>null</startTime>
    <endTime>null</endTime>
    <inferTimeOfIntroduction>false</inferTimeOfIntroduction>
  </analysis>
  <model>
    <epiObservationModel>poisson</epiObservationModel>
  </model>
  <parameters>
    <epiOnly>false</epiOnly>
    <phyloOnly>false</phyloOnly>
    <numParticles>200</numParticles>
    <numSteps>2000</numSteps>
    <numThreads>8</numThreads>
    <numChains>4</numChains>
    <stepCoefficient>0.05</stepCoefficient>
    <resampleEvery>7</resampleEvery>
    <segmentedDays>true</segmentedDays>
    <samplingsAsRemovals>1</samplingsAsRemovals>
    <pairedPsi>false</pairedPsi>
  </parameters>
  <priors>
    <gamma>
      <stepchange>false</stepchange>
      <disttype>TruncatedNormal</disttype>
      <mean>0.15</mean>
      <standarddev>0.05</standarddev>
      <lowerbound>0.0</lowerbound>
   </gamma>
   <psi>
     <stepchange>false</stepchange>
     <disttype>TruncatedNormal</disttype>
     <mean>0.001</mean>
     <standarddev>0.0005</standarddev>
     <lowerbound>0.0</lowerbound>
   </psi>
   <phi>
     <stepchange>false</stepchange>
     <disttype>TruncatedNormal</disttype>
     <mean>0.02</mean>
     <standarddev>0.01</standarddev>
     <lowerbound>0.0</lowerbound>
   </phi>
   <initialBeta>
     <stepchange>false</stepchange>
     <disttype>Uniform</disttype>
     <min>0.3</min>
     <max>0.8</max>
   </initialBeta>
   <betaJitter>
     <stepchange>false</stepchange>
     <disttype>Uniform</disttype>
     <min>0.001</min>
     <max>0.05</max>
   </betaJitter>
  </priors>
</EpiFusionInputs>



We will generate an EpiFusion XML using the summary tree we prepared with the prepare_epifusion_tree function and our loaded case incidence data. First we will make lists of the various parts of the XML file we wish to override from the default. For example, the below code represents the loggers chunk in the default XML that details how often we sample from the MCMC (every 10 MCMC steps):

 <loggers>
   <fileBase>FILESTEM</fileBase>
   <logEvery>10</logEvery>
 </loggers>



To override this, we will make a list in R that we will later pass to the loggers argument of the generate_epifusion_xml function to specify our output folder filepath as Results/fixed_tree and sample from the MCMC chain every 10 steps. We will also make a parameters list to adjust the number of MCMC steps, thus run each chain for longer to ensure we get a satisfactory number of samples from the posterior. We will reduce the number of particles in the particle filter to 100, as this is sufficient for a short, simple analysis, and will slightly reduce runtime.

loggers <- **list**(fileBase = "Results/baseline_fixed_tree", logEvery = 10)
parameters <- **list**(numSteps = 10000,
                   numParticles = 100)



We will also slightly adjust the prior for initialBeta, or $\beta_{0}$ (infectivity at the beginning of the time series) from the default settings. As the default prior for γ is a truncated normal distribution with mean 0.15 , standard deviation 0.05 and lower bound 0.0 , by setting the initial β value as 0.1<β<0.5 we indicate that the initial $R_{t}$ is approximately between 0.66 and 3.33 ( $R_{t}=\beta /\gamma$ , i.e. 0.1/0.15=0.66 and 0.5/0.15=3.33 ).

priors <- **list**(initialBeta = **list**(stepchange = "false",
                            disttype = "Uniform",
                            min = 0.1,
                            max = 0.5))



In this example we are happy with the other parameters in the default XML, so we can generate the XML file Data/EpiFusion_XMLs/fixed_tree_inputfile.xml with the following code:

**generate_epifusion_XML**(tree = "Data/Processed/baseline_fixed_tree.tree",
                       case_incidence = baseline_caseincidence,
                       index_date = index_date,
                       loggers = loggers,
                       priors = priors,
                       parameters = parameters,
                       xml_filepath = "Data/EpiFusion_XMLs/baseline_fixed_tree_inputfile.xml")



**Running EpiFusion**

To run EpiFusion for the fixed tree example, we will use the run_epifusion function from EpiFusionUtilities to run the program within our R session:

**run_epifusion**("Data/EpiFusion_XMLs/baseline_fixed_tree_inputfile.xml")



On conclusion of its analysis, EpiFusion saves a timings.txt file to the output folder with the total runtime in nanoseconds, which we examine and convert to minutes below:

runtime <- **suppressWarnings**(**read.table**("Results/baseline_fixed_tree/timings.txt")[1,1]) **/** 6e10
**paste0**("Runtime: ",runtime," minutes")
## [1] "Runtime: 10.5715153277833 minutes"



**Parsing and plotting the output**

**Figure 4.**
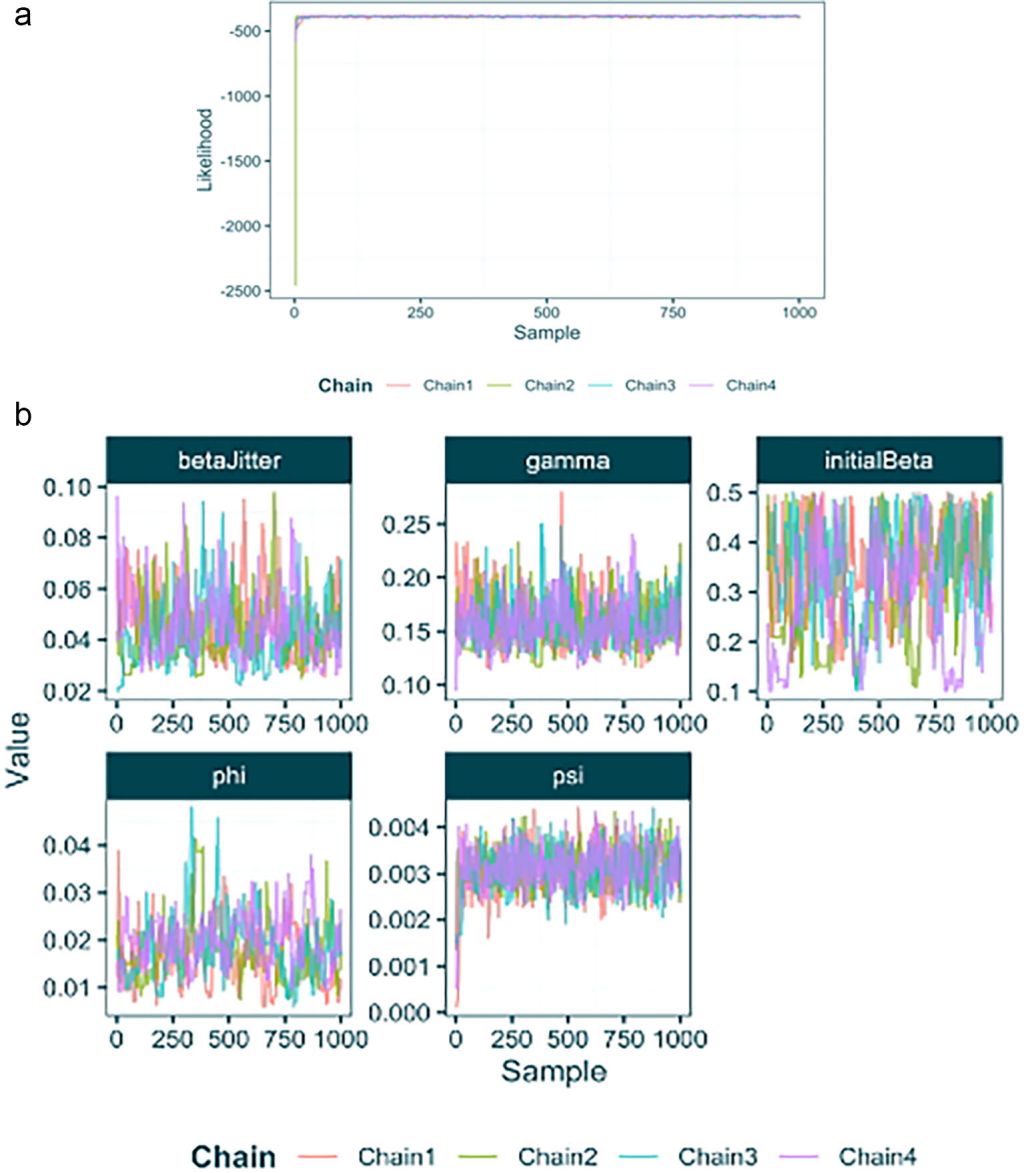
(a) Likelihood and trace plot from an EpiFusion analysis produced by the 'plot_likelihood_trace' function in EpiFusionUtilities. (b) Parameter trace plots from an EpiFusion analysis produced by the 'plot_parameter_trace' function in EpiFusionUtilities.

First we will use the load_raw_epifusion function to import the full raw results. This function automatically produces plots ( Figure 4) of the likelihood and parameter traces using the plot_likelihood_trace and plot_parameter_trace functions. This allows us to check for convergence and help to identify what proportion of each chain to discard as burn-in.

raw_output_fixed <- **load_raw_epifusion**("Results/baseline_fixed_tree/")



Next we can discard the burn-in from each MCMC chain and combine all chains into a combined posterior using the extract_posterior_epifusion function which takes a raw EpiFusion object and the proportion of each chain to discard as burn-in as its arguments. By default, the function returns means and Highest Posterior Density (HPD) intervals for the trajectories and parameters fitted by EpiFusion, however by specifying include_samples = TRUE we also instruct the function to return the actual posterior samples (minus burn-in) for inspection. This greatly increases the memory used by the posterior output object in your R environment, so is recommended for initial inspection of your results but not for downstream tasks such as loading posteriors from many analyses for plotting.

parsed_output_fixed <- **extract_posterior_epifusion**(raw_output_fixed, 0.2, include_samples = TRUE)
**str**(parsed_output_fixed, max.level = 2)
## List of 5
##  $ infection_trajectories : List of 4
##  ..$ mean_infection_trajectory   :  Named num [1:113] 0 1.22 1.54 1.73 1.96 …
##  .. ..- attr(*, "names")= chr [1:113] "T_0" "T_1" "T_2" "T_3" …
##  ..$ median_infection_trajectory : Named num [1:113] 0 1 1 1 2 2 2 2 5 6 …
##  .. ..- attr(*, "names")= chr [1:113] "T_0" "T_1" "T_2" "T_3" …
##  ..$ infection_trajectory_hpdintervals : List of 3
##  ..$ infection_trajectory_samples :'data.frame': 3208 obs. of 113 variables:
## $ rt_trajectories : List of 4
##  ..$ mean_rt_trajectory    :  Named num [1:113] 2.09 2.14 2.18 2.25 2.32 …
##  .. ..- attr(*, "names")= chr [1:113] "T_0" "T_1" "T_2" "T_3" …
##  ..$ median_rt_trajectory  :  Named num [1:113] 2.13 2.16 2.2 2.24 2.32 …
##  .. ..- attr(*, "names")= chr [1:113] "T_0" "T_1" "T_2" "T_3" …
##  ..$ rt_trajectory_hpdintervals : List of 3
##  ..$ rt_trajectory_samples :'data.frame': 3208 obs. of 113 variables:
## $ parameters            :List of 5
##  ..$ gamma      :List of 3
##  ..$ psi        :List of 3
##  ..$ phi        :List of 3
##  ..$ betaJitter :List of 3
##  ..$ initialBeta:List of 3
## $ fitted_epi_cases : List of 4
##  ..$ mean_fitted_epi_cases : Named num [1:15] 0.251 12.306 84.751 297.153 412.487 …
##  .. ..- attr(*, "names")= chr [1:15] "T_0" "T_1" "T_2" "T_3" …
##  ..$ median_fitted_epi_cases : Named num [1:15] 0 12 84 296 412 233 109 51 23 10 …
##  .. ..- attr(*, "names")= chr [1:15] "T_0" "T_1" "T_2" "T_3" …
##  ..$ fitted_epi_cases_hpdintervals : List of 3
##  ..$ fitted_epi_cases_samples :'data.frame': 3208 obs. of 15 variables:
## $ cumulative_infections :List of 4
##  ..$ mean_cuminfection_trajectory : Named num [1:113] 0 0.219 0.542 0.961 1.44 …
##  .. ..- attr(*, "names")= chr [1:113] "T_0" "T_1" "T_2" "T_3" …
##  ..$ median_cuminfection_trajectory   : Named num [1:113] 0 0 0 1 1 2 2 3 6 9 …
##  .. ..- attr(*, "names")= chr [1:113] "T_0" "T_1" "T_2" "T_3" …
##  ..$ cuminfection_trajectory_hpdintervals : List of 3
##  ..$ cuminfection_trajectory_samples :'data.frame': 3208 obs. of 113 variables:



The extracted posterior object from the extract_posterior_epifusion function contains mean and HPD intervals of increasing width for infection, $R_{t}$ , cumulative infection and fitted epidemiological case trajectories. The trajectory_table function can parse these into a convenient table structured to be suitable for plotting with ggplot2. This table is structured with a Time column for each day in the analysis, and Mean and upper and lower HPD interval (0.95, 0.88 and 0.66) columns for each trajectory type (infection, $R_{t}$ , cumulative infections).

traj_table <- **trajectory_table**(parsed_output_fixed, index_date)
**colnames**(traj_table)
##  [1] "Time"                         "Mean_Infected"
##  [3] "Lower95_Infected"             "Upper95_Infected"
##  [5] "Lower88_Infected"             "Upper88_Infected"
##  [7] "Lower66_Infected"             "Upper66_Infected"
##  [9] "Mean_Rt"                      "Lower95_Rt"
## [11] "Upper95_Rt"                   "Lower88_Rt"
## [13] "Upper88_Rt"                   "Lower66_Rt"
## [15] "Upper66_Rt"                   "Mean_CumulativeInfections"
## [17] "Lower95_CumulativeInfections" "Upper95_CumulativeInfections"
## [19] "Lower88_CumulativeInfections" "Upper88_CumulativeInfections"
## [21] "Lower66_CumulativeInfections" "Upper66_CumulativeInfections"

*#Show the first 5 columns and 3 rows of the traj_table*
knitr**::kable**(**head**(traj_table[,1**:**5], n = 3))

**Table:** 

Time	Mean_Infected	Lower95_Infected	Upper95_Infected	Lower88_Infected
2023-12-26	0.000000	0	0	0
2023-12-27	1.218828	1	2	1
2023-12-28	1.542394	1	3	1

**Figure 5.**
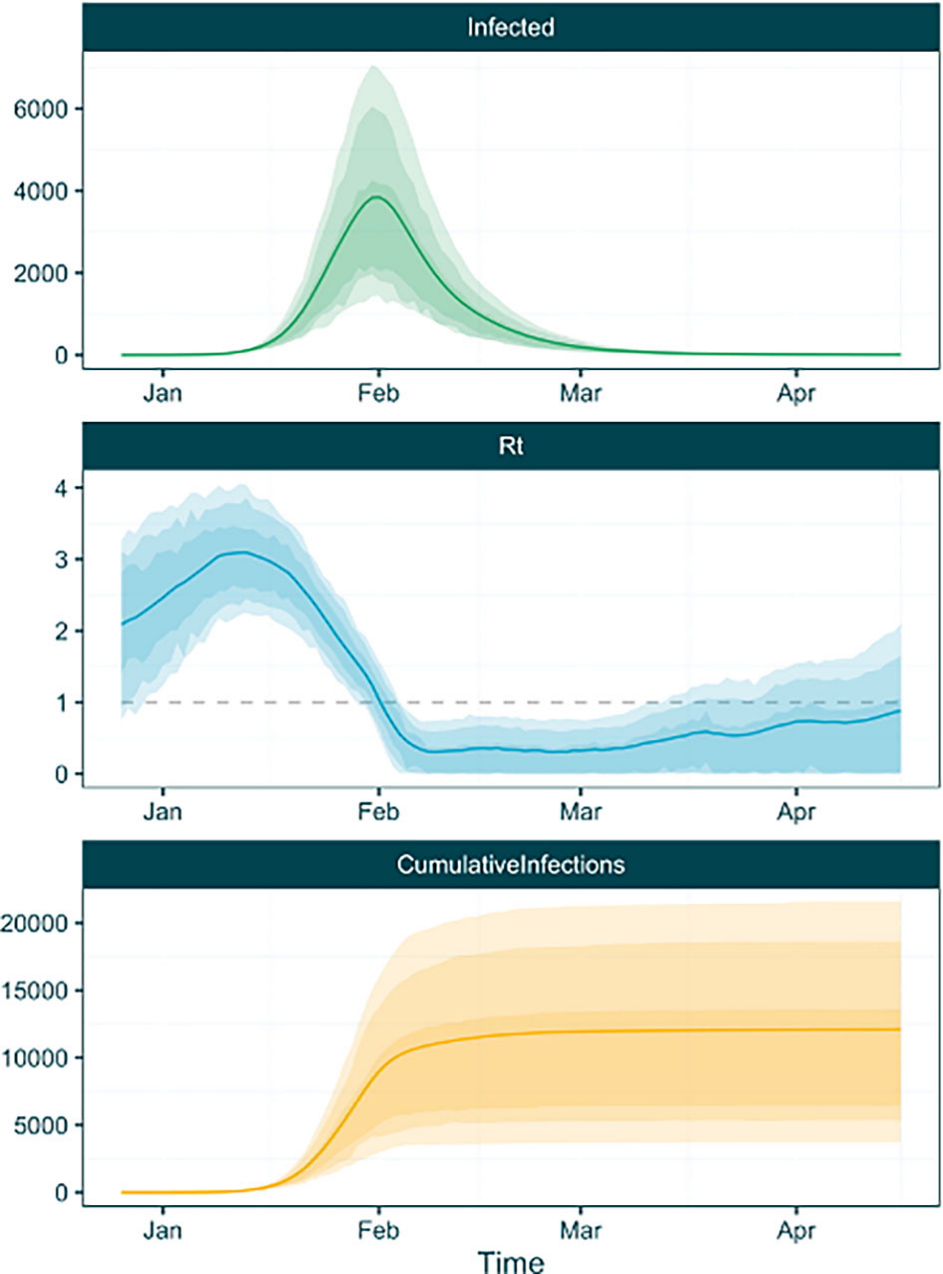
Infection, Rt and cumulative infection trajectories plotted by the EpiFusionUtilities function ‘plot_trajectories’.

It is possible use this table with ggplot functions to plot and inspect the inferred trajectories. However we also provide a function, plot_trajectories that takes the trajectory table as input and automatically plots all three trajectory types ( Figure 5).

**plot_trajectories**(traj_table)

**Figure 6.**
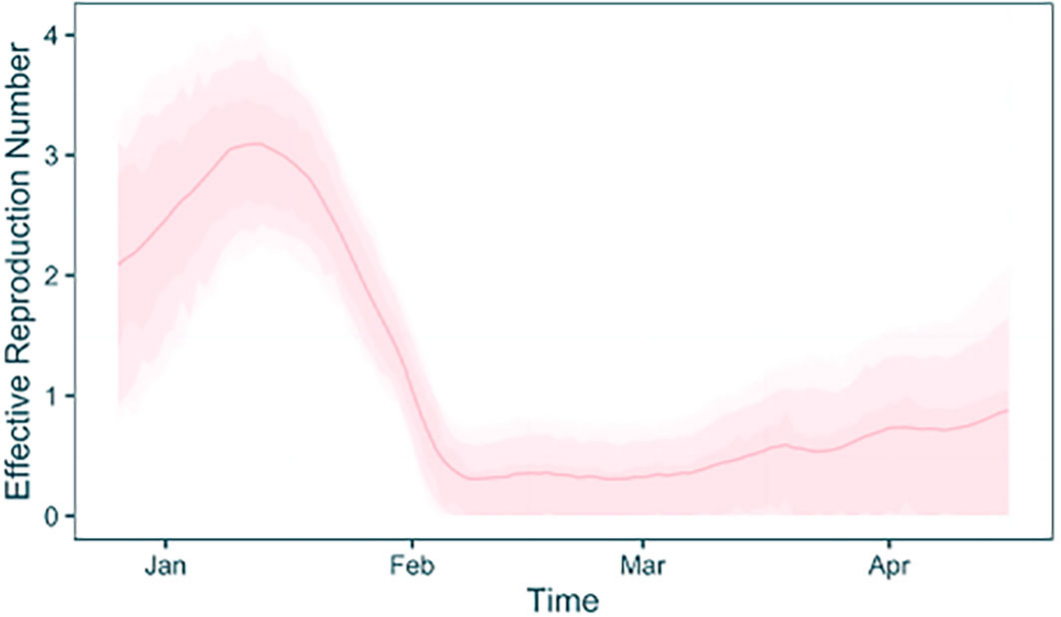
Inferred R(t) trajectories using a combined EpiFusion model and a fixed tree, plotted with the EpiFusionUtilities function ‘plot_trajectories’.

The plot_trajectories function also takes additional arguments to allow more customisation. For example, it is possible to provide a specific trajectory type to plot using the type argument, and specify bespoke plot colours using the plot_colours argument. Here we will plot only the $R_{t}$ trajectories in a specified colour (pink) ( Figure 6).

**plot_trajectories**(traj_table, type = "rt", plot_colours = "pink")

**Figure 7.**
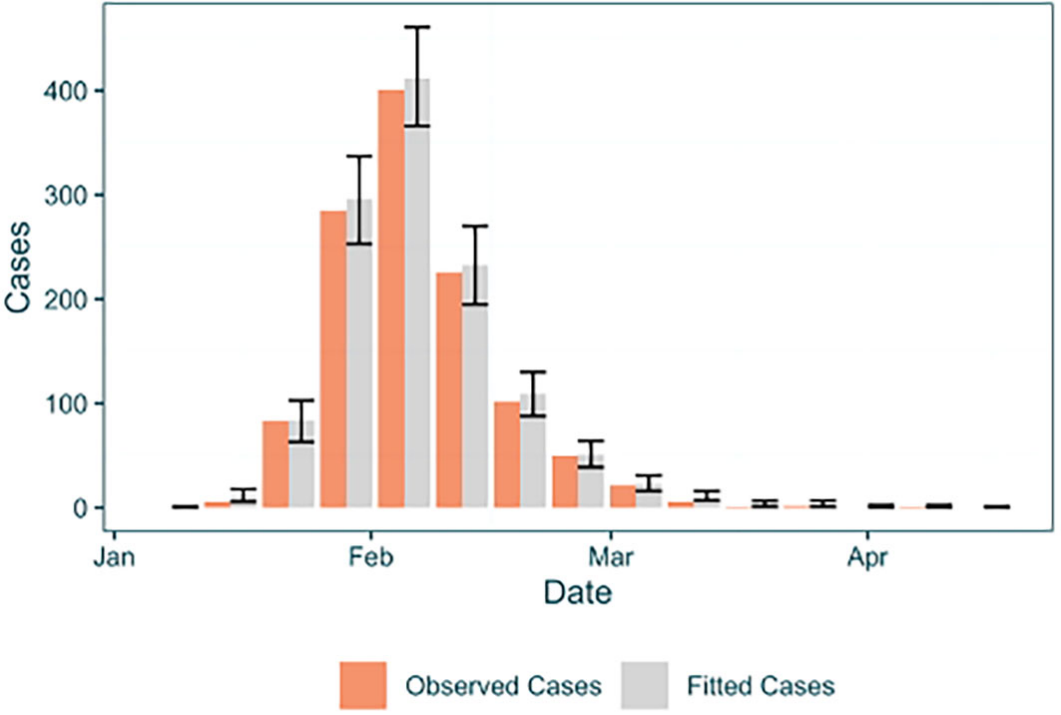
Fit of observed epidemiological cases to simulated cases by the EpiFusion model, plotted with ggplot2.

As this was a combined analysis that has used case incidence data, it is possible to examine the fit of the case incidence simulated within the model to the provided data. We already have the case incidence data loaded from the data preparation stage, so we can add the mean and HPD intervals of the fit to the existing table ( Figure 7).

epi_data_and_fit_table <- baseline_caseincidence **%>%**
  **mutate**(Stat = "Observed Cases") **%>%**
  **full_join**(**data.frame**(Date = baseline_caseincidence**$**Date,
                       Stat = "Fitted Cases",
                   Cases = parsed_output_fixed**$**fitted_epi_cases**$**median_fitted_epi_cases,
                   Lower95_Cases = parsed_output_fixed**$**fitted_epi_cases**$**fitted_epi_cases_hpdintervals**$**HPD0.95**$**Lower,
                   Upper95_Cases = parsed_output_fixed**$**fitted_epi_cases**$**fitted_epi_cases_hpdintervals**$**HPD0.95**$**Upper
         )) **%>%**
  **mutate**(Stat = **factor**(Stat, levels = **c**("Observed Cases", "Fitted Cases")))
## Joining with ‘by = join_by(Cases, Date, Stat)’
**ggplot**(epi_data_and_fit_table, **aes**(x = Date)) **+**
  **geom_bar**(**aes**(y = Cases, fill = Stat), stat = "identity", position = "dodge", col = NA, alpha = 0.7) **+**
  **scale_fill_manual**(name = "", values = **c**("#e95b0d", "grey")) **+**
  **geom_errorbar**(**aes**(ymin = Lower95_Cases, ymax = Upper95_Cases, col = Stat), position = "dodge", show.legend = F) **+**
  **scale_color_manual**(values = **c**(NA, "black")) **+**
  **lshtm_theme**()



Observed epidemiological cases are shown by the grey bars, with their corresponding fitted cases from EpiFusion shown to their right by the blue bars. The error bars for the fitted case incidence correspond to bounds of the 0.95 HPD interval.

Finally we can examine the posteriors of the MCMC parameters. The posterior extraction process uses the R package stable.GR to perform gelman-rubin convergence tests on each parameter, and estimate the effective sample sizes of each. If the gelman-rubin statistic is less than 1.015 this indicates MCMC convergence. ^18^ If the MCMC has not converged it may be necessary to run each chain for longer.

**print**(parsed_output_fixed**$**parameters**$**gamma**$**rhat)
## [1] 1.001274
**print**(parsed_output_fixed**$**parameters**$**gamma**$**ess)
## [1] 1052



**Figure 8.**
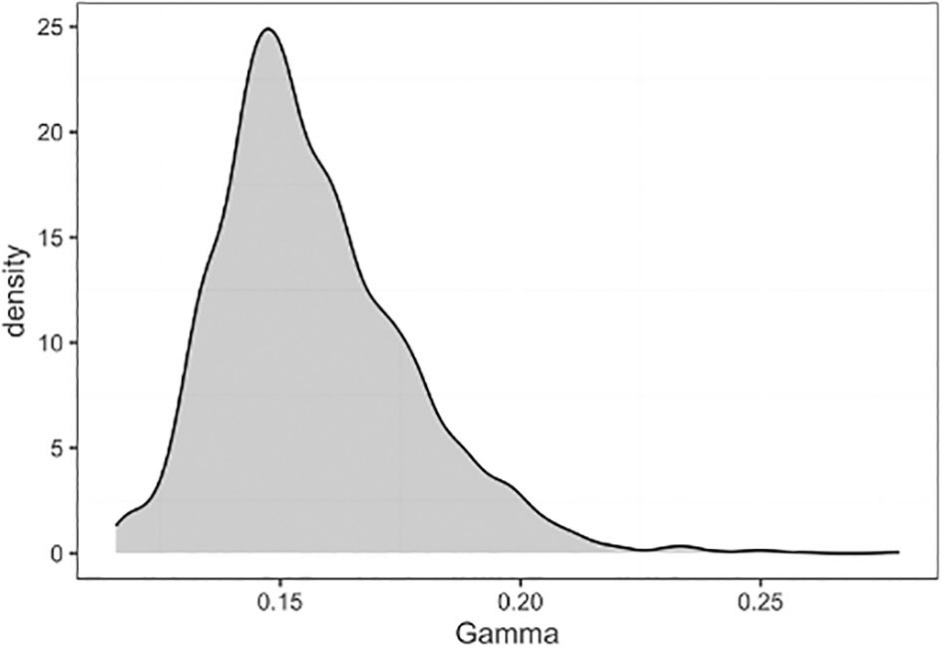
Posterior density of the gamma recovery/removal parameter, plotted using ggplot2.

We can also view the posterior density of a parameter by plotting the samples from the MCMC, which we can access from the posterior object due to setting include_samples = TRUE when we extracted the posterior earlier using extract_epifusion_posterior ( Figure 8).

**ggplot**(data = **data.frame**(Gamma = parsed_output_fixed**$**parameters**$**gamma**$**samples), **aes**(x = Gamma)) **+**
  **geom_density**(fill = "#01454f", alpha = 0.3) **+**
  **lshtm_theme**()

### Phylogenetic uncertainty

In the previous example we modelled the baseline outbreak dataset using a fixed time-scaled phylogenetic tree as the phylodynamic data source. However, in a real outbreak setting there is often uncertainty about the evolutionary relationships and hence the tree sturcture (phylogenetic uncertainty). Bayesian tree inference approaches such as BEAST ^22, 24^ attempt to approximate the true tree by sampling trees from a posterior set obtained through MCMC, often yielding thousands of plausible tree structures under the provided data and model. A single maximum clade credibility tree can be summarised from this ‘tree posterior’ and was used in EpiFusion using workflow specified above. It is valuable, however, to assess how uncertainty in the tree structure may affect epidemiological parameters inferred through EpiFusion. Currently, this can be explored by using a tree posterior as the data input into EpiFusion, and sampling a unique tree from the posterior for use with each MCMC chain. Below we demonstrate this approach by once more modelling the baseline outbreak dataset, but this time using the tree posterior as the phylodynamic data source.

**Prepare data and parameters and run EpiFusion**

To prepare the tree posterior (which was already loaded into our environment when we used the baseline_dataset() function) we once again use the prepare_epifusion_tree function. This function will recognise that a tree posterior has been passed, and will write the processed trees to a file without returning anything to your R session.

**prepare_epifusion_tree**(baseline_treeposterior, index_date, last_sequence, "Data/Processed/baseline_processed_tree_posterior.tree")



Next we will generate the XML file for the analysis using the tree posterior. We again specify adjustments to the loggers chunk, specifying our desired output folder name and how often to sample from the MCMC and print to console. We will also increase the number of MCMC chains to 50, which, in conjunction with passing a tree posterior to EpiFusion, will instruct the model to run 50 chains, each using a different tree sampled at random from the tree posterior. This analysis will therefore take longer.

loggers <- **list**(fileBase = "Results/baseline_tree_posterior", logEvery = 5)parameters <- **list**(numChains = 50)



Similarly to the fixed tree example, we will adjust some of the default priors. As before, we will set the initial infectivity $\beta_{0}$ to between 0.1 and 0.5 . We will also narrow slightly narrow some priors using their inferred values as estimated from our previous example on the same dataset, in order to help the efficiency of the MCMC sampling process due to the extra number of chains we are running.

priors <- **list**(initialBeta = **list**(stepchange = "false",
                                  disttype = "Uniform",
                                  min = 0.1,
                                  max = 0.5),
               betaJitter = **list**(stepchange = "false",
                                  disttype = "Uniform",
                                  min = 0.005,
                                  max = 0.05),
               phi = **list**(stepchange = "false",
                                  disttype = "TruncatedNormal",
                                  lowerbound = 0.0,
                                  mean = 0.02,
                                  standarddev = 0.005))



Finally we generate an XML file using these parameters and priors for input into EpiFusion and run it:

**generate_epifusion_XML**(tree = "Data/Processed/baseline_processed_tree_posterior.tree",
                       case_incidence = baseline_caseincidence,
                       index_date = index_date,
                       loggers = loggers,
                       parameters = parameters,
                       priors = priors,
                       xml_filepath = "Data/EpiFusion_XMLs/tree_posterior_inputfile.xml")

**run_epifusion**("Data/EpiFusion_XMLs/tree_posterior_inputfile.xml")



**Inspecting each chain**

To examine the results of the model using the tree posterior we will again load the raw results with load_raw_epifusion . This time we will set suppress_plots to true.

raw_phylouncertainty <- **load_raw_epifusion**("Results/baseline_tree_posterior/", suppress_plots = TRUE)



**Figure 9.**
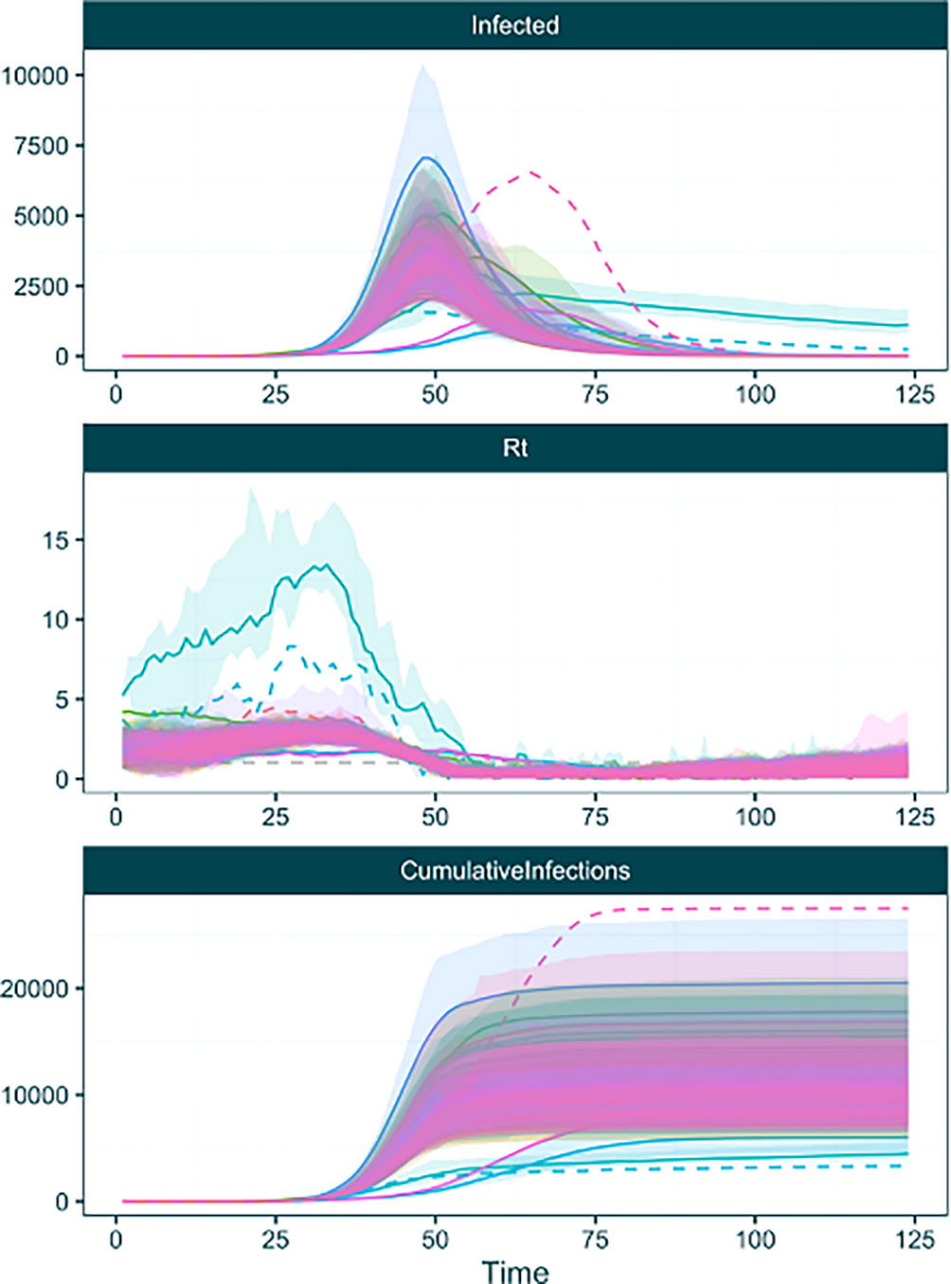
Inferred infection, R _t_ and cumulative infection trajectories plotted using the ‘plot_chainwise_trajectories’ function of EpiFusionUtilities.

To examine the effect the inclusion of the tree posterior has on the analysis, we can use another EpiFusionUtilities function plot_chainwise_trajectories . This function operates similarly to the plot_trajectories function, but separates the trajectories by chain for inspection, while discarding a proportion of the trajectories of each chain for burn-in. This allows us to see how the sampled tree, which differs between each chain, affects the inferred trajectories ( Figure 9). Here we can see that most of the chains converge on a similar set of trajectories to our fixed tree analysis, but some chains (and thus, some sampled trees) suggest other trajectory possibilities.

**plot_chainwise_trajectories**(raw_phylouncertainty, 0.2)
## [1] "WARNING: Chain 1 got stuck, with an acceptance rate of 0.0."
## [1] "WARNING: Chain 34 got stuck, with an acceptance rate of 0.0."
## [1] "WARNING: Chain 50 got stuck, with an acceptance rate of 0.0."

In this plot we see a further capability of the plot_chainwise_trajectories function. MCMC chains that have become ‘stuck’ i.e. enter a state space where they do not accept any further MCMC steps and have an acceptance rate of 0% are plotted with dotted lines, to enable users to identify and discard them when extracting the posterior using the discard_chains argument of the extract_posterior_epifusion function.

**Figure 10.**
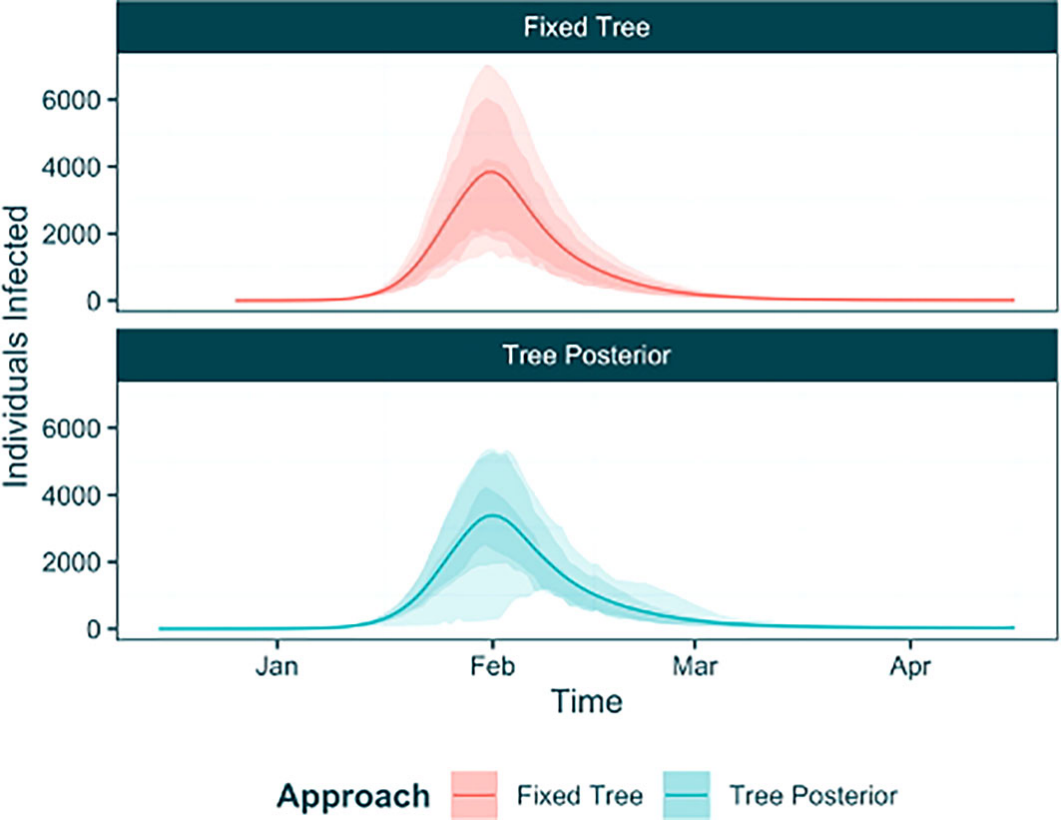
Inferred infection trajectories from EpiFusion analyses using a fixed tree (red) vs a tree posterior (blue).

When we extract the posterior from our raw output object (while discarding the ‘stuck’ chains), the chains will be combined and this uncertainty will be represented in our posterior estimates. To further understand this uncertainty we will extract the posterior sample using the extract_posterior_epifusion function and again create a trajectory table using the trajectory_table function. Using this table, and our trajectory table from the fixed tree analysis, we can use ggplot2 to plot the trajectories from both analyses to demonstrate the effect of the phylogenetic uncertainty on the estimates ( Figure 10). The tree posterior approach is characterised by a widening of the HPD intervals around the mean fitted infection trajectory, due to the phylogenetic uncertainty.

posterior_phylouncertainty <- **extract_posterior_epifusion**(raw_phylouncertainty, 0.3, discard_chains = **c**(1, 34, 50))
phylouncertainty_trajtable <- **trajectory_table**(posterior_phylouncertainty, **as.Date**("2023-12-15")) **%>%**
  **mutate**(Approach = "Tree Posterior")

combined_trajtable <- traj_table **%>%**
  **mutate**(Approach = "Fixed Tree") **%>%**
  **rbind**(phylouncertainty_trajtable)

**ggplot**(combined_trajtable, **aes**(x = Time, col = Approach, fill = Approach)) **+**
  **geom_line**(**aes**(y = Mean_Infected)) **+**
  **geom_ribbon**(**aes**(ymin = Lower95_Infected, ymax = Upper95_Infected), col = NA, alpha = 0.2) **+**
  **geom_ribbon**(**aes**(ymin = Lower88_Infected, ymax = Upper88_Infected), col = NA, alpha = 0.2) **+**
  **geom_ribbon**(**aes**(ymin = Lower66_Infected, ymax = Upper66_Infected), col = NA, alpha = 0.2) **+**
  **lshtm_theme**() **+**
  **labs**(y = "Individuals Infected") **+**
  **facet_wrap**(**~**Approach, ncol = 1)

This approach to combining chains sampled under different phylogenetic trees to form unified posteriors should be carefully employed. For the purposes of demonstration here we sample 50 trees in unique chains, however to adequately approximate the full tree posterior it is advised to conduct more samples. Further, if there is significant disparity in the inferred trajectories from different chains (i.e. under different trees), we recommend reexamining the tree posterior to check for overt phylodynamic uncertainty in your tree data and considering whether employing EpiFusion is suitable with highly uncertain phylogenies.

### Introducing rate changes

While our previous examples with the baseline dataset describe an outbreak with constant sampling throughout, real life scenarios are often more complicated as the rates that govern our model, β , γ , ϕ and ψ vary over time. β is allowed to vary over time by default and is fit in the particle filter, but below we will address a common scenario where rates of sampling cases and genomes ( ϕ and ψ ) increase sharply at a given time point. For example, this occurred during the Brazilian Zika outbreak in 2015, where sampling sharply increased following the introduction of widespread PCR testing. ^25^

**Data preparation**

We can load the data for this example using the EpiFusionUtilities function sampling_dataset(). We will use the same index date for this analysis as previously, but for this dataset the last sequence in the tree was sampled on March 17th, so we will adjust the ‘last_sequence’ date accordingly. As in our other examples, we prepare our tree data for EpiFusion using the prepare_epifusion_tree function.

**sampling_dataset**()
**print**(sampling_caseincidence[1**:**5,])
**print**(sampling_tree)

last_sequence <- **as.Date**("2024-03-17")

sampling_fixed_tree <- **prepare_epifusion_tree**(sampling_tree, index_date, last_sequence, "Data/Processed/sampling_fixed_tree.tree")



**Advanced parameterisation: Time variant prior distributions**

In this example we wish to parameterise the step-increase in sampling on February 5th in our model. We will do this by setting a ‘time variant prior’ for case sampling rate phi when we generate the XML file, and using the ‘paired psi’ feature ( *Supplementary Information Appendix 3*) to pair the genomic sampling rate psi to the case sampling rate.

Previously in the EpiFusion input files, the phi block in the prior section consisted of the following XML code:

   <phi>
       <stepchange>false</stepchange>
       <disttype>TruncatedNormal</disttype>
       <mean>0.02</mean>
       <standarddev>0.01</standarddev>
       <lowerbound>0.0</lowerbound>
   </phi>



A phi parameter with a step change is adjusted to look like this:

   <phi>
     <stepchange>true</stepchange>
     <changetime>
       <x0>
         <disttype>FixedParameter</disttype>
         <value>35</value>
       </x0>
     </changetime>
     <distribs>
       <x0>
         <disttype>TruncatedNormal</disttype>
         <mean>0.002</mean>
         <standarddev>0.0001</standarddev>
         <lowerbound>0.0</lowerbound>
       </x0>
       <x1>
         <disttype>TruncatedNormal</disttype>
         <mean>0.025</mean>
         <standarddev>0.005</standarddev>
         <lowerbound>0.0</lowerbound>
       </x1>
     </distribs>
   </phi>



The key differences here include the setting of the stepchange parameter to true, and the introduction of two new sub-nodes, changetimes and distribs, that contain the prior distribution details for the times of the rate changes in days from the index date (changetimes), and the rates themselves ( distribs). For a rate with n change points, there must be n+1 distributions in distribs and n distributions in changetimes. These distributions are provided in tags with the format xn. While these adjustments can be made manually, it is also possible to parameterise this through the priors argument of generate_epifusion_xml using nested lists.

First we will make a list of the phi changetimes (in this example there is only one). In this scenario we ‘know’ the date of the step change in sampling - February 5th, 41 days after our index date - so we will provide it as a fixed parameter. However it is feasible to infer this change, if desired, by providing any discrete non-fixed prior distribution for this parameter.

phi_changetimes <- **list**(x0 = **list**(disttype = "FixedParameter",
                                  value = 41))



Next we will provide prior distributions for phi before and after the provided change time in the distribs.

phi_distribs <- **list**(x0 = **list**(disttype = "TruncatedNormal",
                               mean = 0.005,
                               standarddev = 0.002,
                               lowerbound = 0.0),
                     x1 = **list**(disttype = "TruncatedNormal",
                               mean = 0.05,
                               standarddev = 0.02,
                               lowerbound = 0.0))



The list structure we introduce below using the changetimes and distribs we have created mirrors the structure of the XML chunk.

phi_prior <- **list**(stepchange = "true",
             changetime = phi_changetimes,
             distribs = phi_distribs)



We can then feed this to the priors argument when we generate the XML file. We also will set pairedPsi to true in the parameters, and provide an empty pairedPsi in the priors. This specifies that psi is not to be fit by MCMC, and the genomic sampling rate psi is calculated as a proportion of the case sampling rate using the proportion of genomic sequences to cases in the data. Further information on this process is available in the Supplementary Information.

**generate_epifusion_XML**(tree = "Data/Processed/sampling_fixed_tree.tree",
                       case_incidence = sampling_caseincidence,
                       index_date = index_date,
                       loggers = **list**(fileBase = "Results/sampling_step_change", logEvery = 5),
                       parameters = **list**(pairedPsi = "true",
                                         numSteps = 10000),
                       priors = **list**(phi = phi_prior,
                                     pairedPsi = ""),
                       xml_filepath = "Data/EpiFusion_XMLs/sampling_fixed_tree_inputfile.xml")

**run_epifusion**("Data/EpiFusion_XMLs/sampling_fixed_tree_inputfile.xml")



Pairing psi with phi in this way is optional; here we couple the rates as we know they should change at the same time. It is also possible to parameterise these separately, e.g. an increase in sequencing without a corresponding increase in case sampling.

**Parsing results**

**Figure 11.**
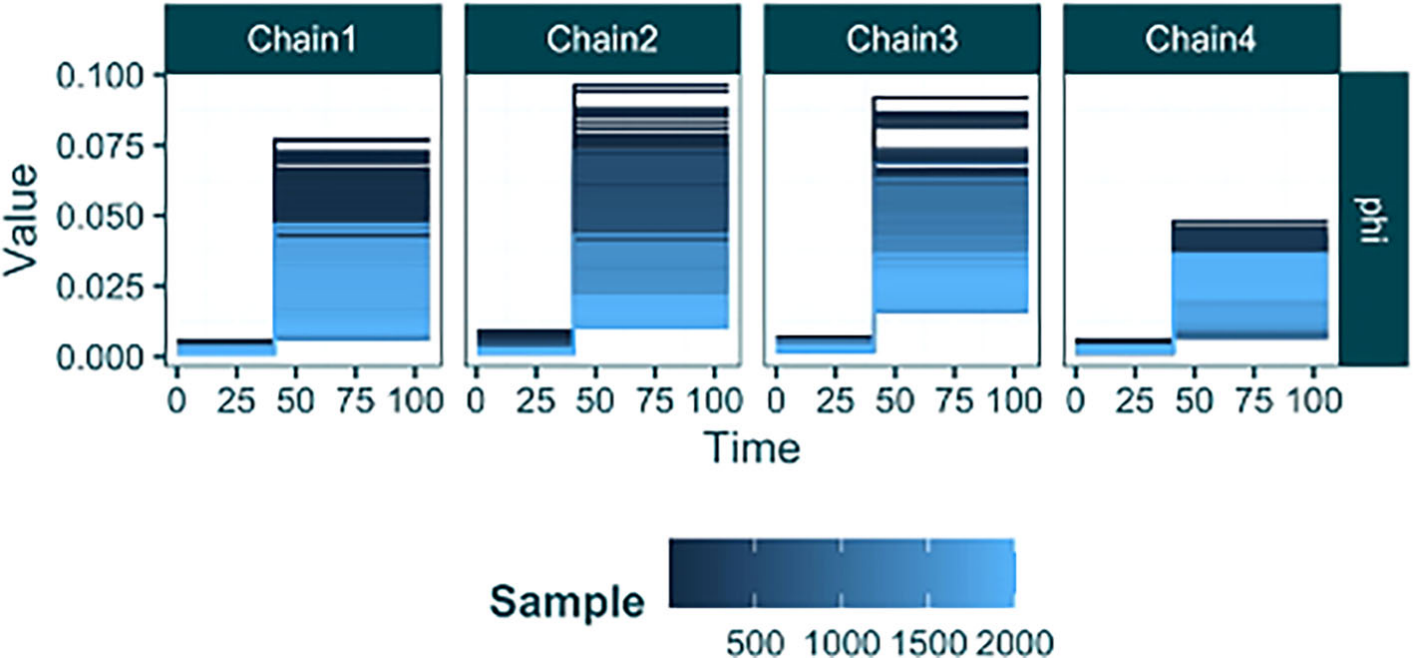
Parameter traces from an EpiFusion analysis with a case sampling rate ( phi ) step change.

To complete our analysis we will load our results using the load_raw_epifusion function once more, and inspect the parameter trace. Here we will suppress the automatically created plots, and specifically plot the parameter trace of interest (the time varying parameters) using the plot_parameter_trace function, but changing the default type from all to timevar ( Figure 11).

raw_sampling <- **load_raw_epifusion**("Results/sampling_step_change/", suppress_plots = TRUE)
**plot_parameter_trace**(raw_sampling, type = "timevar")



Here the output from plot_parameter_trace looks slightly different to previous versions (e.g. Figure 4). The function automatically recognises the presence of parameters that vary over time, and plots their piecewise constant values (y-axis) across time (x-axis) in step graphs. This allows the inferred value over time to be intuitively understood from the plot. The lines are coloured by their sample index on a continuous gradient, making visible the values to which the each chain has converged (light blue). Here we see that despite each chain initiating at different values, the initial and final sampling rates across each chain converge to approximately the same values. This is shown by the light blue (later MCMC samples) lines occurring at the same y-axis value in each chain trace plot for the phi parameter.

The parsing and plotting process for the rest of the results from this analysis follows the same steps as the other vignettes included in this article.

## Conclusions and Discussion

The EpiFusion Analysis Framework is a novel workflow for implementing the EpiFusion joint epidemiological and phylodynamic inference model using the Java implementation of the model and the R package EpiFusionUtilities. This workflow is generalisable, utilising common R objects for data formatting ( data.frame and phylo or multiPhylo objects) and parameterisation (list objects). We detail the full analysis workflow here, including a new feature introduced since EpiFusion’s first description: introduction of the ability to explore the effect phylogenetic uncertainty by providing a tree posterior as data.

Use of this approach is recommended for outbreaks where a time-scaled phylogenetic tree and case incidence data is available, and the desired result is continuous (to a daily resolution) models of pertinent outbreak trajectories such as $R_{t}$ and infections over time. The data sampled should arise from the same ‘outbreak system’; specifically the genomic sequences used to build the tree and the case data should be sampled from the same approximate geographic location, time period, and population. If a time-scaled phylogenetic tree is not already assembled, this may be generated from established phylodynamic tree estimation approaches such as BEAST ^22^ or Nextstrain. ^26^ The program runs efficiently for even very large trees, but run-time increases with the length of the time series under investigation, ^10^ accordingly we currently do not recommend EpiFusion for analyses of time periods of longer than five years. In these instances, other programs such as EpiNow2 ^27^ (case incidence data only) or TimTam ^28, 29^ (both genomic and case incidence data), written in R and Java (BEAST Framework) respectively, may be more appropriate.

The documentation of reproducible analysis workflows, particularly for new tools, is essential for open research. ^7– 9^ Providing efficient pipelines with corresponding instructions enables researchers to build on previous work to address empirical research in an efficient way, which can be of great importance during outbreak settings which are often time sensitive. ^30^ In the three vignettes described we provide examples of standard EpiFusion parameterisation, however there are many advanced options available to users to customise their analysis. These include capabilities for composite (non-parametric) prior distributions ( *Supplementary Information Appendix 4)*, multiple epidemiological observation model options ( *Supplementary Information Appendix 2, ‘Model’*), multiple options for fitting $\beta_{t}$ ( *Supplementary Information Appendix 5*), and buffer zones for rate step-changes ( *Supplementary Information Appendix 6*).

Exploration of the effect of phylogenetic uncertainty is now incorporated in the program through allowing the use of a time-rooted phylogenetic tree posterior (from a software such as BEAST) to be used as data within the model. This new feature is an implementation rather than theoretical advancement: For each unique MCMC chain, a new tree is randomly sampled from this posterior and used as the tree data in the model. The resulting posteriors can subsequently be examined and combined by the user with post-hoc EpiFusionUtilities functions. Incorporating phylogenetic uncertainty predictably led to increased uncertainty in the model estimates in the combined posterior. We encourage care in implementing this approach, and thoroughly examining the effect of using different trees on the model estimates using the plot_parameter_trace and plot_chainwise_trajectories functions. Further, for efficiency in our example shown above, we sample 50 trees, whereas it may be necessary to increase this number to adequately approximate the tree posterior. Going forward we aim to investigate other approaches for incorporating phylogenetic uncertainty in the model in a more comprehensive manner.

To demonstrate the advanced parameterisation options of the framework, we addressed an outbreak with a step-increase in case and genomic sequence sampling rates. While this example featured a single change in the modelled rates, this infrastructure is very flexible and can be used to add significant complexity to the model according to the user requirements.

The current framework is robust, but has some limitations. Traditionally R packages such as EpiFusionUtilities are distributed using the R package ecosystem CRAN. However, CRAN does not accept packages which contain binary executable code, ^31^ so it would not have been possible to distribute an EpiFusion release (an executable jar file) with the package and enable running the model from within R. A likely future step in the software development process may be to fully integrate the model into the EpiFusionUtilities R package using R/Rcpp, to allow more universal usage for all users including those without a extensive phylodynamic experience and would enable the package to be hosted on CRAN.

The requirement to provide a user defined index date, or earliest possible date of outbreak origin, is a practical implementation compromise that may result in incorrect conclusions if the index date is not set early enough (i.e., if the index date is accidentally set after the true date of outbreak origin). Currently this can be overcome by setting the index date to a longer time period before the suspected origin of the outbreak, however the resulting estimates during the earlier periods of the modelled time series should be treated carefully and will typically display high levels of uncertainty. Future distributions of this framework will aim to allow inference without this truncation to a specified index date, or allow the index date to be inferred within the model.

In conclusion, this article aims to outline a reproducible framework for utilising our novel joint inference model using a functional R package and a binary executable file. We show how different parameterisations and options for the analysis can be implemented, including how to introduce phylogenetic uncertainty through the provided tree data, and time-variant prior distributions. We hope that clearly outlining a use case of the framework will facilitate its implementation by researchers to investigate hypotheses of public health importance in the future.

## Ethics and consent

Ethical approval and consent were not required.

## Data Availability

For the purposes of open research, we make available all code and data used in this manuscript on GitHub. The source code for EpiFusion is written in Java and available on GitHub at the following repository: https://github.com/ciarajudge/EpiFusion. The source code is made available under the GNU General Public License (v3) ( https://www.gnu.org/licenses/gpl-3.0.en.html). The source code at the time of this publication is archived on Zenodo: https://doi.org/10.5281/zenodo.14973874 ^32^ The source code for EpiFusionUtilities is written in R and is available on GitHub at the following repository: https://github.com/ciarajudge/EpiFusionUtilities. The source code is made available under the GNU General Public License (v3) ( https://www.gnu.org/licenses/gpl-3.0.en.html). The source code at the time of this publication is archived on Zenodo: https://doi.org/10.5281/zenodo.14973878 ^33^ The simulated datasets used in this manuscript have been described in Section 4 and are provided in the Open Science Framework repository ‘EpiFusion Analysis Framework Software Article’: https://doi.org/10.17605/OSF.IO/7W43Y. ^34^ The project contains the following underlying data:
• baseline_caseindicence.RDS: weekly case incidence from the baseline simulated dataset (R data.frame) • baseline_treeposterior.RDS: posterior of time-scaled phylogenetic trees from the baseline simulated outbreak dataset (R S3 multiPhylo object) • baseline_tree.RDS: time-scaled phylogenetic tree from the baseline simulated outbreak dataset (R S3 phylo object) • sampling_caseindicence.RDS: weekly case incidence from the step-change in sampling simulated dataset (R data.frame) • sampling_tree.RDS: time-scaled phylogenetic tree from the step-change in sampling simulated outbreak dataset (R S3 phylo object) Data are made available under the terms of the Creative Commons Attribution 4.0 International Licence (CC-BY 4.0) ( https://creativecommons.org/licenses/by/4.0/). The code used to produce all analyses and plots in this manuscript is available on GitHub at the following repository: https://github.com/ciarajudge/EpiFusion_Vignettes. This repository is archived on Zenodo: https://doi.org/10.5281/zenodo.14973889. ^35^ Data and code are made available under the terms of the Creative Commons Attribution 4.0 International Licence (CC-BY 4.0) ( https://creativecommons.org/licenses/by/4.0/). The extended data in this manuscript (additional figures, tables and appendices) is available as a Supplementary Information file which is provided in the Open Science Framework repository ‘EpiFusion Analysis Framework Software Article’: https://doi.org/10.17605/OSF.IO/7W43Y. ^34^ The project contains the following extended data:
• supplementary_information.pdf: PDF file containing Appendices 1-6 with supplementary information.
