# Supplementary material for: The EpiFusion Analysis Framework for joint phylodynamic and epidemiological analysis of outbreak characteristics
Source: F1000Res. 2025 Mar 28;14:345. [Version 1] doi: 10.12688/f1000research.162719.1 (PMC12134729; doi:10.12688/f1000research.162719.1)
Supplement: Peer review history

# Reviewer report: Reviewer response for version 1

**DOI:** 10.5256/f1000research.178965.r376911

**Published:** 2025-06-03

**Authors:** Leo A Featherstone

**Affiliations:** 1 The University of Melbourne, Melbourne, Victoria, Australia

**Overview**

 Judge et al. present the EpiFusionUtilities R package. It provides an R interface for the EpiFusion program implemented in java, but with the key extension to accommodating phylogenetic uncertainty. This is a valuable contribution both in providing a means to address phylogenetic uncertainty and by improving accesibility through an R interface. The examples provided are comprehensive and explained with good clarity. 

**Some points for clarification**

 P6: " *It is also possible to test multiple extractions extract_posterior_epifusion() with different burn-in proportions, and inspect the gelman-rubin statistics of the parameters, which indicate convergence if below 1.015*"

 It would be helpful to outline how the Gelman-Rubin statistic relates to effective sample size (ESS), which will be more familiar to BEAST users who appear to be part of the intended user base here. Could users equivalently read output into the `coda` R package to calculate ESS for MCMC traces?

 P16: " *...It is valuable, however, to assess how uncertainty in the tree structure may affect epidemiological parameters inferred through EpiFusion. Currently, this can be explored by using a tree posterior as the data input into EpiFusion, and sampling a unique tree from the posterior for use with each MCMC chain...*"

 Does the input strictly have to be a posterior distribution of trees? I gather the program is useful for comparing the effects of any set of candidate trees in a multiPhylo object, which I think is a really useful feature!

 P22 *Figure 11*:

 I'm concerned that any patterns in colour could be obscured by overlapping of states. Would you consider faceting by sub-groupings of samples or perhaps implementing some version of a parallel coordinates plot to better tease apart states? Here's an example: (https://beastiary.wytamma.com/plots/parallel-coordinates/) from the beastiary [1] program.

 P22: " *The program runs efficiently for even very large trees, but run-time increases with the length of the time series under investigation,10 accordingly we currently do not recommend EpiFusion for analyses of time periods of longer than five years*"

 It sounds like the number of data points in the time series is the issue rather than the total timespan. If so, can you provide an upper limit on the workable number of rows in the time series? E.g. I assume 5 years of monthly or weekly time series data would be better than daily?

 P23: " *Currently this can be overcome by setting the index date to a longer time period before the suspected origin of the outbreak, however the resulting estimates during the earlier periods of the modelled time series should be treated carefully and will typically display high levels of uncertainty. Future distributions of this framework will aim to allow inference without this truncation to a specified index date, or allow the index date to be inferred within the model.*"

 Please elaborate on how overly conservative index dates affect inference. Is it correct to intuit that having very old index date would deflate estimates of transmission rate?  How would this interact with the 5-year time limit you proposed above?

 P17: Typo: " *We will also narrow slightly narrow some priors*"

Are the conclusions about the tool and its performance adequately supported by the findings presented in the article?

Yes

Is the rationale for developing the new software tool clearly explained?

Yes

Is the description of the software tool technically sound?

Yes

Are sufficient details of the code, methods and analysis (if applicable) provided to allow replication of the software development and its use by others?

Yes

Is sufficient information provided to allow interpretation of the expected output datasets and any results generated using the tool?

Yes

Reviewer Expertise:

Phylodynamics; Phylogenetics

I confirm that I have read this submission and believe that I have an appropriate level of expertise to confirm that it is of an acceptable scientific standard.

## References

[1] : Real-Time and Remote MCMC Trace Inspection with Beastiary. *Molecular Biology and Evolution* .2022;39(5) : 10.1093/molbev/msac095 10.1093/molbev/msac095 PMC915603535552742

# Reviewer report: Reviewer response for version 1

**DOI:** 10.5256/f1000research.178965.r376910

**Published:** 2025-05-15

**Authors:** Claire Guinat

**Affiliations:** 1 Interactions Hôtes-Agents Pathogènes (IHAP), Université de Toulouse, INRAE, Toulouse, France

**Overall comment**

 This documentation is very relevant to facilitate the correct use of the EpiFusion tool and is essential for open research. However, the text and code should be harmonized/complemented by what is also presented in the online tutorial on the GitHub page. The first part, which I think aims to be more generic, is a bit confusing; it becomes clearer when we reach the applications. I ran the different parts of the code, which run smoothly. However, here are a few points that might improve the manuscript:

  

- I also went to https://github.com/ciarajudge/EpiFusion/tree/main/examples
  => What are the links between the three tutorials provided on GitHub and this paper? The connection is unclear. It would be helpful to integrate all sources of information or clarify their respective purposes to avoid fragmentation.
- Table 1 – About Data
  => Is the tree time-calibrated? Please clarify whether this refers to an MCC tree or a posterior sample of time-scalibrated trees.
- About Parameters
  => The term “parameters” usually refers to model parameters. Here, it appears to refer to inference parameters or hyperparameters. Please clarify this distinction.
- About Model
  => What are the available model options? The XML shows a Poisson model, but further explanation is needed on what each model represents (e.g., incidence process), and how to choose one over another.
- Add library(EpiFusionUtilities) after devtools::install_github(...)
  => This addition would improve clarity and ensure reproducibility for users unfamiliar with the package.
- Index_case <- as.Date(“2024-01-01”)
  => If this date cannot be estimated and must be fixed, what are the implications? Could you clarify how users should handle uncertainty, perhaps by suggesting a sensitivity analysis? Also, is this date just an example or related to real data?
- “If there is uncertainty in the date of origin of the outbreak we recommend setting the index date to earlier than the estimated date.”
  => Please clarify what is meant by "estimated date"—is it based on genetic data, case reports, or another source?
- “To prepare a tree or posterior set of trees for EpiFusion”
  => The documentation should clarify whether trees must be time-calibrated. This is crucial for users preparing their data.
- prepare_epifusion_tree(...)
  => Are users expected to use the tree provided in the GitHub tutorial? Providing a simple MCC tree as an example would be helpful for learning purposes.
- “This function populates a template XML...”
  => It would help to list default parameter values and priors explicitly here. Also, clarify terminology: "priors" and "parameters" typically refer to biological model settings, whereas here they seem to relate to the inference framework (MCMC, particle filtering, etc.).
- CSV case format for incidence data
  => Please give an explicit description of the required format (e.g., columns, headers). You could refer to the full workflow tutorial but be explicit here too.
- generate_epifusion_XML(...)
  => The full workflow tutorial uses a different example and mentions priors, but it's unclear which parameters are being referred to. I recommend listing all adjustable priors (e.g., ψ, ϕ, betajitter) and defining them clearly.
- run_epifusion("epifusion_input.xml")
  => Could you include an estimate of run time or a way to monitor progress, similar to BEAST logs? This would help users plan their analyses.
- Output files (betas.csv, params.txt, acceptance.txt)
  => Clarify that params_chain0.csv is the output referenced as params.txt. Are these MCMC parameters or model parameters? Define what the “acceptance” file contains and how to interpret acceptance rates.
- “It is possible to process this raw output manually...”
  => Before visualizing outputs, users should be advised to assess convergence (e.g., trace plots, Gelman-Rubin statistics). Mention this step earlier in the process.
- Rt estimates
  => Where are the Rt estimates? Can users specify whether Rt is estimated from tree only, case data only, or combined? Clarifying this would be very useful.
- Use cases section
  => The term “Use cases” might be misleading, since “case” is already used for case incidence data. Perhaps “Applications” would be a clearer section title.
- “Finally, in ‘Introducing Rate Changes’”
  => Consider rewording as “Introducing sampling rate changes” to be more specific.
- Structured populations
  => Is it possible to use the method for structured populations and estimate different Rt values per subpopulation? Clarify whether this is supported or planned.
- “To generate the data...” using ReMaster.19
  => Do we assume that all cases are reported? Clarify whether the incidence file reflects total or sampled incidence, since under-reporting is common in real-world outbreaks.
- “...csv file with dated counts of weekly incidence”
  => Does incidence have to be weekly? Could it be daily or in another time unit? Specify how to align tree and case data temporal scales.
- Methods vs. Use Cases
  => The “Use Cases” section is much clearer than the “Methods” section. Consider stating explicitly that the methods part is a generic overview and that detailed application examples follow.
- include_samples = TRUE
  => It is unclear what changes when this is set to TRUE. Does it apply burn-in and return a trimmed posterior sample? Please clarify the effect of this option.
- parsed_output_fixed <- extract_posterior_epifusion(...)
  => If users want to compute their own summary statistics (e.g., medians), should they use the raw output after burn-in? Clarify the recommended procedure.
- “Finally we can examine the posteriors of the MCMC parameters...”
  => Introduce convergence checks (Gelman-Rubin, ESS) earlier in the workflow. Also, define acronyms like ESS and rhat, and explain what these values represent and how to interpret them.
- print(parsed_output_fixed$parameters$gamma$rhat) output
  => It’s not clear to beginners what this output means. Relate it clearly to the earlier explanation of convergence diagnostics.
- “However, in a real outbreak setting there is often uncertainty...”
  => Typo: "sturcture" should be "structure".
- “...sampled at random from the tree posterior...”
  => Please clarify: if multiple trees are sampled, does this represent a sensitivity analysis across tree uncertainty? The phrasing earlier in the document suggests that only one tree is used per run.
- “...run 50 chains, each using a different tree sampled...”
  => This is now clearer, thanks. It might help to emphasize that this is the way to explore tree uncertainty.
- Side question
  => I wonder why the authors of the original EpiFusion paper are not included in this manuscript? Their inclusion would make sense if the method is being described or re-implemented.

Are the conclusions about the tool and its performance adequately supported by the findings presented in the article?

Yes

Is the rationale for developing the new software tool clearly explained?

Yes

Is the description of the software tool technically sound?

Yes

Are sufficient details of the code, methods and analysis (if applicable) provided to allow replication of the software development and its use by others?

Partly

Is sufficient information provided to allow interpretation of the expected output datasets and any results generated using the tool?

Partly

Reviewer Expertise:

epidemiology, infectious diseases, transmission dynamics, phylodynamics

I confirm that I have read this submission and believe that I have an appropriate level of expertise to confirm that it is of an acceptable scientific standard, however I have significant reservations, as outlined above.

# Reviewer report: Reviewer response for version 1

**DOI:** 10.5256/f1000research.178965.r374093

**Published:** 2025-05-07

**Authors:** Vijaykrishna Dhanasekaran, Ruopeng Xie

**Affiliations:** 1 The University of Hong Kong, Hong Kong, Hong Kong

This manuscript provides a valuable practical extension of the EpiFusion framework, offering clear guidance for implementing joint phylodynamic-epidemiological analyses via the EpiFusionUtilities R package. The inclusion of vignettes and real-world outbreak case studies significantly enhances reproducibility and accessibility, bridging the gap between theoretical models and applied public health research. Below are suggestions to further strengthen the work:

 1.    The emphasis on reproducibility is a major strength. To ensure seamless replication across systems, the authors could:

 ·       Encapsulate dependencies by providing Docker containers or platform-agnostic virtual environments (e.g., via renv).

 ·       Document software versions explicitly, including a sessionInfo() output, R/Java version requirements, and a requirements.txt file (or equivalent) to help users mirror the analysis environment.

 2.    While the focus on EpiFusionUtilities is appropriate, the manuscript would benefit from a comparative discussion of how EpiFusion complements or differs from established tools (e.g., BEAST, PhyDyn, Outbreaker2). A concise table or paragraph highlighting unique features (e.g., joint modeling of genomic and epidemiological data) and limitations (e.g., computational scalability) would help users assess its suitability for their needs.

 3.    The manuscript includes analyses of two outbreak datasets. Briefly justify their selection and relevance to contemporary public health challenges to strengthen the narrative. A more explicit discussion of the framework’s assumptions and limitations would improve its practical utility:

 ·       The model assumes a single infected individual (single index) initiates the outbreak. Discuss its sensitivity to this assumption and whether the model can accommodate multiple introductions.

 ·       While the manuscript notes that phylogenetic uncertainty affects parameter estimation, it should also address how variation in tree-building methods (e.g., ML vs. Bayesian approaches) might propagate into epidemiological inferences.

 ·       Guidance on computational performance (e.g., runtime benchmarks for larger datasets or complex models) would help users gauge feasibility for their own work.

Are the conclusions about the tool and its performance adequately supported by the findings presented in the article?

Yes

Is the rationale for developing the new software tool clearly explained?

Yes

Is the description of the software tool technically sound?

Yes

Are sufficient details of the code, methods and analysis (if applicable) provided to allow replication of the software development and its use by others?

Yes

Is sufficient information provided to allow interpretation of the expected output datasets and any results generated using the tool?

Yes

Reviewer Expertise:

NA

We confirm that we have read this submission and believe that we have an appropriate level of expertise to confirm that it is of an acceptable scientific standard.
